# Experienced pain after stroke: a cross-sectional 5-year follow-up study

**DOI:** 10.1186/s12883-019-1584-z

**Published:** 2020-01-07

**Authors:** Emma Westerlind, Ramanjit Singh, Hanna C. Persson, Katharina S. Sunnerhagen

**Affiliations:** Department of Clinical Neuroscience, Institute of Neuroscience and Physiology, University of Gothenburg, Per Dubbsgatan 14, 413 45 Gothenburg, Sweden

**Keywords:** Stroke, Pain, Quality of life, Follow-up

## Abstract

**Background:**

Stroke is one of the most common cause of disability worldwide. Pain is common in both stroke survivors and in the general population. Consequences of post-stroke pain (PSP) include reduced quality of life and are important to consider. The aim of the current study was to explore the experience of pain 5 years after stroke, and factors associated with the experience of pain.

**Methods:**

Inclusion criteria were: First ever stroke, treated at Sahlgrenska University Hospital, Sweden, during an 18 months period in 2009–2010, aged 18 years or older. Furthermore, the participants had to respond to a set of questionnaires 5 years post-stroke. Baseline data were collected from medical records and follow-up data from the set of questionnaires. The primary outcome was based on the question *Do you experience pain?* Predictors and explanatory factors for experiencing more frequent pain were analysed with logistic regression.

**Results:**

A total of 281 participants were included. Almost 40% experienced pain to some degree 5 years post-stroke (15% reported pain frequently), and 25% felt that their needs for pain treatment were not met. The participants experiencing more frequent pain reported poorer quality of life, self-perceived health status and recovery post-stroke. Functional dependency at discharge from hospital, experiencing depression at follow up and restricted mobility at follow up were all associated with more frequent pain.

**Conclusion:**

Pain is common 5 years post-stroke and the treatment is not perceived as optimal. The persons experiencing more frequent pain seem to rate their health and recovery worse than the persons experiencing less frequent pain. Most of the factors associated with more frequent pain were treatable and this emphasize the importance of standardised follow-up care that takes pain into consideration.

## Background

Stroke is the second most common cause of death [1], though due to improved preventative health care and an ageing population the number of people surviving a stroke is increasing [2]. The growing population of stroke survivors may experience a variety of complications such as depression, physical disability, cognitive impairment and post-stroke pain (PSP) [3].

The prevalence of pain as reported in research varies, but a large study in the US showed that more than 50% of a general population had experienced pain the last 3 months [4]. Consequences of pain include reduced quality of life [5], reduced capacity to take part in daily activities [6], interference with occupation [7] and depressive disorders [8].

Pain as a consequence after stroke has a prevalence of 11–66% [9]. However, pain is not always directly correlated to stroke and data from several studies suggest that PSP is more common in patients with pain prior to the stroke [10, 11]. There are several different types of PSP such as headache, shoulder pain, pain due to muscle stiffness, spasm, complex regional pain syndrome and central PSP [12–14]. This may also include experience of pain and presence of sensory abnormalities in body parts affected by the cerebrovascular lesion [12].

A review study presented that risk factors of PSP included female sex, older age, alcohol use, and depression [9]. Furthermore, stroke-related risk factors of PSP included ischemic stroke, spasticity, reduced upper extremity movement and sensory deficits [9].

Most previous studies have focused on specific types of pain and few have investigated the prevalence of any type of PSP [15]. Furthermore, the time to pain assessment after stroke varies in previous research, but follow-up more than 2 years post-stroke is uncommon [9, 16]. PSP affects daily living [17] and can be an obstacle to rehabilitation [18]. Additional research is therefore of great importance in order to provide the optimal health-care for the effected individuals.

The aim of the current study was to explore experience of pain 5 years after stroke, and factors associated with the experience of pain.

## Methods

### Study design and participants

This cross-sectional follow-up study was based on data from the Stroke Arm Longitudinal study at the University of Gothenburg (SALGOT)-extended [19–21]. During 18 months between February 2009 and December 2010 all patients admitted to a stroke unit, neurosurgical ward or intensive care unit at Sahlgrenska University Hospital in Gothenburg, Sweden, were eligible for the SALGOT-extended study. The inclusion criteria were: first-ever ischemic stroke (IS) (I63), intracerebral haemorrhage (ICH) (I61) or subarachnoid haemorrhage (SAH) (I60) diagnosis; resident in the Gothenburg urban area (within 35 km from the Sahlgrenska University Hospital); be 18 years old or older at stroke onset. The Sahlgrenska University Hospital is the single centre in the area that provides interventions such as thrombectomy and thrombolysis.

Data from the acute phase was collected from medical records. Five years post-stroke, the survivors received a postal survey (including two reminders) consisting of a set of questionnaires focusing on the long-term consequences of stroke. The set of questionnaires included a follow-up questionnaire from Riksstroke, EuroQol Quality of life scale (EQ5D) and the Stroke Impact Scale (SIS).

### Clinical assessments

At arrival to hospital, stroke severity was (in the case of IS or ICH) assessed with National Institutes of Health Stroke Scale (NIHSS) 0–46 [22] and in patients with confirmed SAH with Hunt and Hess (H&H) 1–5, where a lower score suggests a less clinically severe presentation [23]. The NIHSS total score was divided into the following groups: very mild (0–2), mild (3–4), moderate (5–15) and severe (16-46). At discharge from hospital, the functional dependency was assessed with the modified Rankin Scale (mRS) 0–5 [24]. The mRS was dichotomized in the current study into: functionally independent if score 0–2 and functionally dependent if score 3–5 [25]. From medical records at stroke onset, two chronic pain-related comorbidity variables were collected; musculoskeletal disorders and all possible pain-related disorders. Musculoskeletal disorders included for instance; neck pain, arthrosis, and rheumatoid arthritis. Pain-related conditions included all the musculoskeletal disorders as well as conditions such as migraine, angina pectoris, and cancer.

### Questionnaire follow-up

From the Riksstroke questionnaire, the questions *what is your level of mobility, do you feel depressed, do you experience pain,* and *have your needs of treatment for pain been met* were used. The question about depression was dichotomized into experiencing depression (answering *often* or *always*) or not (answering *never/almost never* or *sometimes*). The level of mobility was dichotomized into intact mobility (answering: *mobile indoors and outdoors without assistance*) and restricted mobility (*needing assistance outdoors* or *always needing assistance*). The experience of pain was dichotomized into experiencing less pain (answering *never/almost never* or *sometimes*) and experiencing more pain (answering *often* or *always*).

The EQ5D [26] was used to measure health-related quality of life. The EQ5D includes questions relating to the following domains; mobility, self-care, usual activities, pain/discomfort and anxiety/depression. Each of the questions can be answered as no problem, some problem or extreme problem. In addition to this, a visual analogue scale (VAS) estimate the respondents self-perceived health ranging from 0 to 100 (higher score corresponds to less self-perceived health problems).

The stroke Impact Scale (SIS) [27, 28] is used to measure post-stroke recovery. In the present study, a VAS was used to provide an estimate of the respondents’ recovery ranging from 0 to 100 (higher score is better) were used.

### Statistical analysis

All analyses were carried out using the IBM SPSS version 23. Significance level was set at *p* < 0.05. Differences between groups were analysed with Fischer’s exact test and Mann Whitney U test.

Logistic regression was used to investigate predictors and explanatory factors for experiencing more pain. Two separate regression models, one predictive and one explanatory, were built. Dependent variable in both models were the dichotomized experience of pain. Independent variables in the predictive model were: age at stroke onset, sex, the dichotomized functional dependency at discharge, existence of musculoskeletal disorders, and existence of all possible pain-related disorders. Independent variables in the explanatory model were: age at time of follow-up, sex, the dichotomized functional dependency at discharge, existence of musculoskeletal disorders and existence of all possible pain-related disorders, the dichotomized level of mobility and the dichotomized experience of depression.

In the model building procedure, an adequate sample size was first ensured. Multicollinearity between independent variables was investigated using Spearman’s correlation test with correlation coefficients <±0.7 being acceptable for inclusion in the regression model. Univariate logistic regressions were performed for each independent variable with a *p*-value < 0.25 considered acceptable for inclusion in the multivariable model. In the multivariable analysis, backward stepwise selection was used with the significance level set at *p* < 0.05. Finally, variables excluded in univariate analysis were re-inserted to the final model one by one to and included if statistically significant. The goodness of fit and the accuracy of the multivariable regression models were evaluated with Hosmer and Lemeshow Test, the Nagelkerke R square and a Receiver Operating Characteristic (ROC) curve [29]. An area under the ROC curve > 0.70 indicate acceptable accuracy.

## Results

**Fig. 1:**
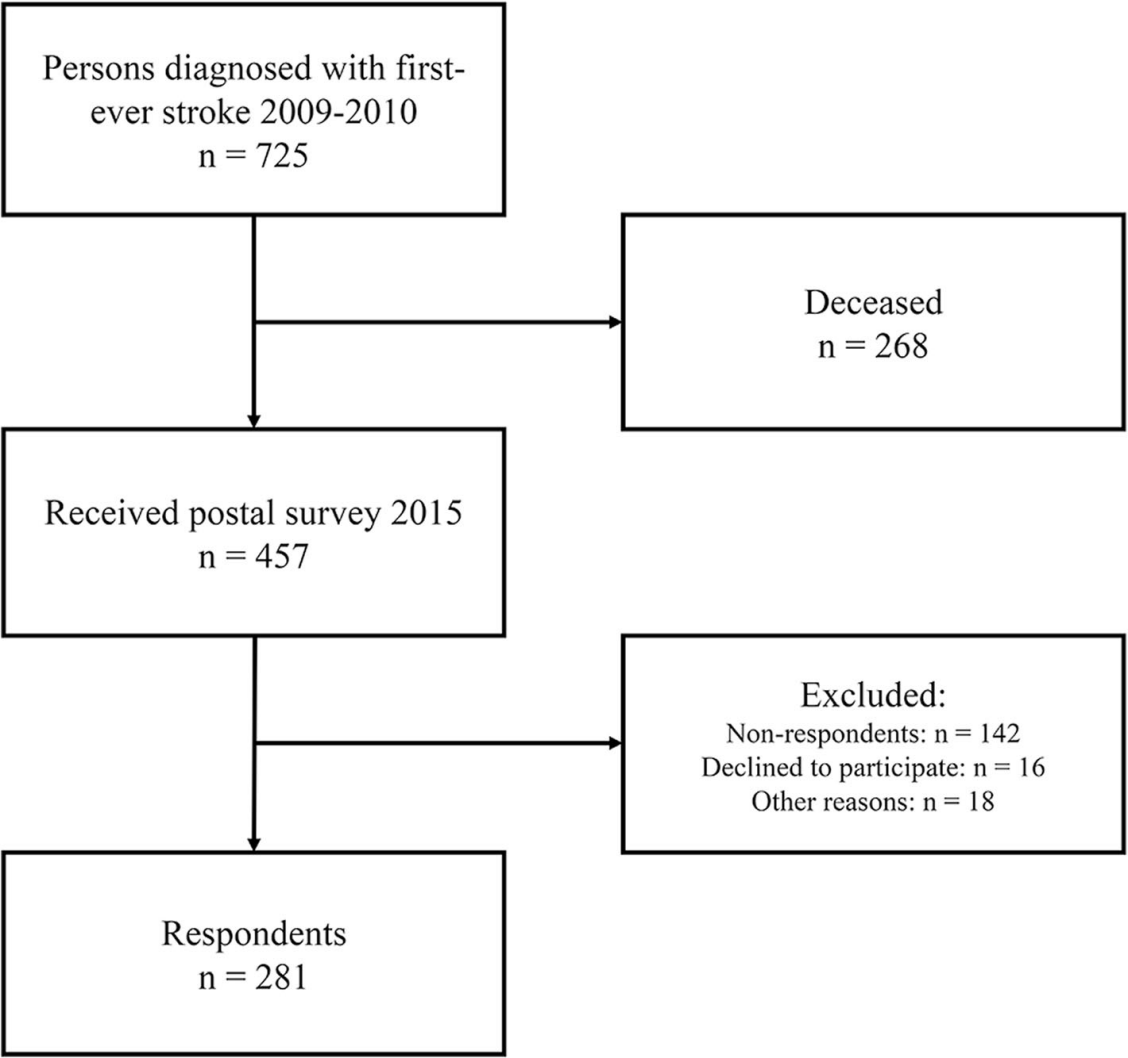
Flowchart of the inclusion of the participants

As seen in Fig. 1, of the 457 persons that received the mail survey, 281 participants responded. There was no significant difference between respondents and non-respondents regarding level of functional dependency at discharge from hospital or in age. However, differences were seen regarding sex, the non-respondents consist of more women than men (*p* = 0.001).

The mean age at stroke onset was 65 years and 61% of the participants were men (Table 1). Less than 7% of the participants were dependent in personal activities of daily living (ADL) and approximately 25% were dependent in instrumental ADL five years post-stroke.

**Table 1:** The participants characteristics at stroke onset and at five years post-stroke (*n* = 281)

Demographics	
Age at time of stroke onset, mean (SD)	65.4 (13.5)
Sex, n (%)	
Male	171 (60.9)
Female	110 (39.1)
Stroke type, n (%)	
IS	218 (77.6)
ICH	36 (12.8)
SAH	27 (9.6)
NIHSS at stroke onset, n (%), *n = 230*	
Very mild (0–2)	144 (62.6)
Mild (3–4)	25 (10.9)
Moderate (5–15)	46 (20.0)
Severe (16–46)	15 (6.5)
Median (min-max)	1 (0–24)
H&H, n (%), *n = 27*	
Grade 1	4 (14.8)
Grade 2	14 (51.9)
Grade 3	2 (7.4)
Grade 4	4 (14.8)
Grade 5	1 (3.7)
Unknown	2 (7.4)
Comorbidity, n (%), *n = 276*	
Musculoskeletal disorder	36 (13.0)
Pain-related disorders	76 (27.5)
Characteristics five years post-stroke	
Do you receive help with eating/drinking? n (%) *n = 277*	
Yes	4 (1.4)
Do you receive help going to the bathroom? n (%) *n = 279*	
Yes	15 (5.4)
Do you receive help getting dressed? n (%), *n = 278*	
Yes	19 (6.8)
Do you receive help with grocery shopping? n (%), *n = 279*	
Yes	73 (26.2)
Do you receive help with cleaning? n (%), *n = 279*	
Yes	117 (41.9)
What is your level of mobility? n (%), *n = 279*	
Intact mobility	250 (89.6)
Restricted mobility	29 (10.4)
Do you feel depressed? n (%), *n = 270*	
Experiencing depression	38 (14.1)
Not experiencing depression	232 (85.9)
Do you feel pain? n (%), *n = 271*	
Experiencing more pain	42 (15.5)
Experiencing less pain	229 (84.5)
Self-perceived recovery post-stroke (SIS), median (min-max), *n = 261*	80 (0–100)
Self-perceived health status post-stroke (EQ VAS), median (min-max), *n = 254*	80 (10–100)

Abbreviations: *IS* Ischaemic stroke, *ICH* Intracerebral haemorrhage, *SAH* Subarachnoid haemorrhage, *NHISS* National Institutes of Health Stroke Scale, *H&H* Hunt and Hess, *SIS* Stroke Impact Scale, *EQ-VAS* EuroQol Quality of life scale- Visual analogue scale

### Experienced pain

Of the 271 patients who answered the question *do you experience pain* 19 (7%) reported always having pain, 23 (9%) often, 66 (24%) sometimes, and 163 (60%) never/almost never. Dichotomized, this gives 15% (42 persons) that reported more frequent pain and 85% (229 persons) that reported less frequent pain. Participants who reported having more frequent pain also reported experienced depression (*p*-value < 0.001) and restricted mobility (p-value = 0.002) compared to participants who had less frequent pain. Furthermore, participants that were functional dependent at discharge also reported more frequent pain (p- value = 0.018). There were no statistically significant differences between the groups of participants with more or less frequent pain regarding age at stroke onset, sex, comorbidity, living conditions or prior history of stroke. Participants with more frequent pain scored their self-perceived health status and recovery post-stroke significantly lower (median value 50 and 55 respectively) in comparison with participants with less frequent pain (median value 80 and 85 respectively), *p*-value < 0.001.

**Fig. 2:**
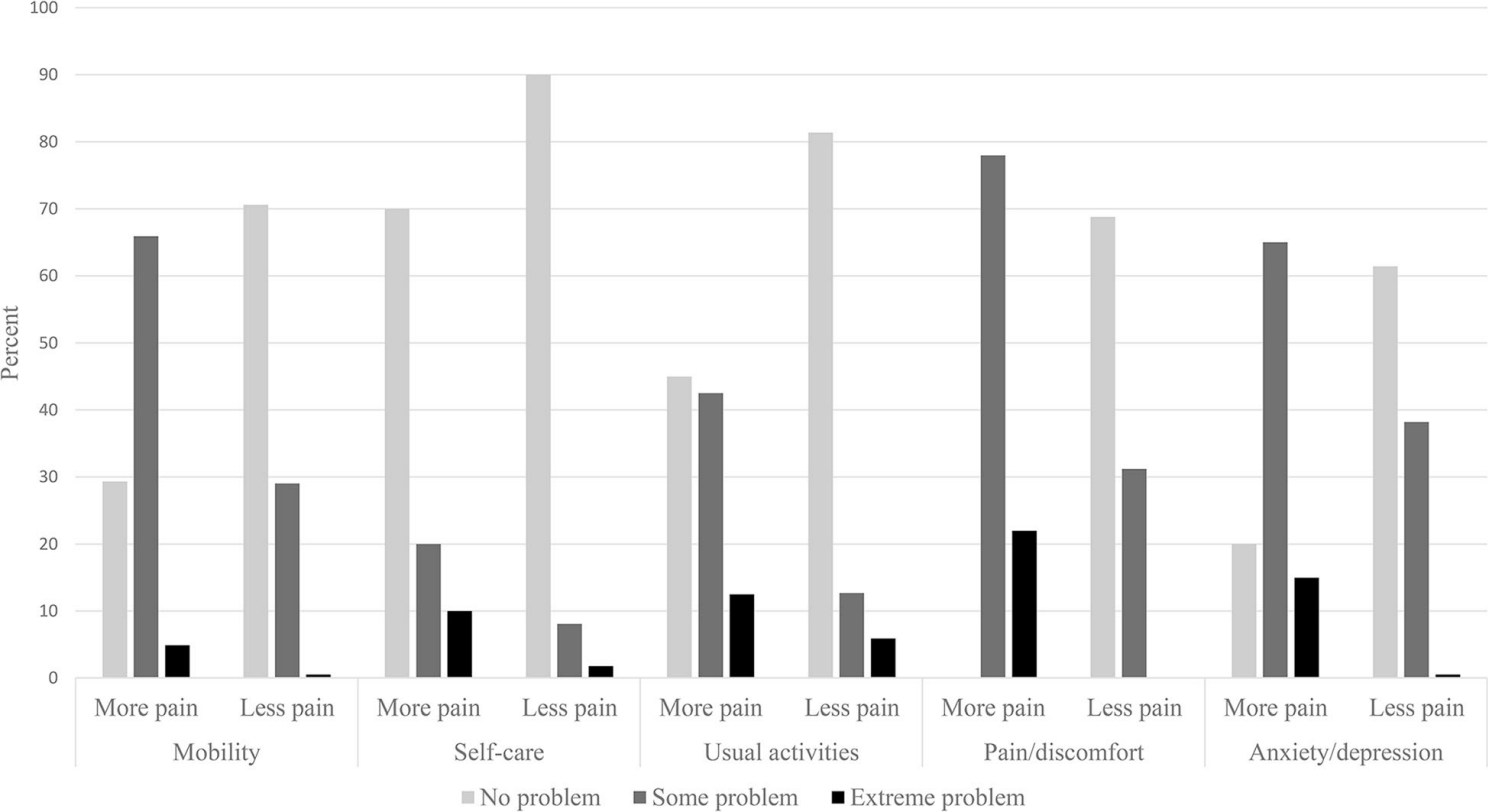
Health-related quality of life in the EQ5D domains, compared between the 2 pain groups

The EQ5D domains compared between the pain groups are presented in Fig. 2. More frequent pain was associated with poorer outcome in all five domains (*p*-value < 0.001).

Of the 269 participants responding to the question about pain treatment, 64% reported having no need for pain treatment, 11% were fully satisfied with their treatment while 19 and 6% reported that their needs were only partially or not at all met respectively. There were no significant differences between participants with fulfilled needs and those not satisfied with received treatment regarding sex, age, comorbidity.

### Factors associated with pain

The participants that were functionally dependent at discharge from hospital, compared to the functionally independent had higher odds of experiencing more frequent pain 5 years post-stroke (OR 2.413) in the predictor model (Table 2). Age, musculoskeletal disorders and pain-related disorders did not fulfil statistical criteria, and were not included in the model. Of the included factors, sex did not contribute significantly to the final model.

**Table 2:** Significant predictors of post-stroke pain in multivariable analysis

Predictor variables	*P*-value	OR	95% C.I. for OR
Functionally dependent	0.014	2.413	1.194–4.878

Area under the ROC-curve 0.60. Nagelkerke R Square 0.041. Abbreviations: OR: odds ratio; C.I: confidence interval

The results from the explanatory multivariable regression analysis are presented in Table 3. Age, musculoskeletal disorders and pain-related disorders were excluded prior to the final regression model due to unfulfilled statistical criteria, and sex and level of functional dependency did not make a significant contribution to the model. Having restricted mobility (OR 3.649) and experiencing depression (OR 7.953) were significant explanatory factors for more frequent pain.

**Table 3:** Significant explanatory variables of post-stroke pain in multivariable analysis

Explanatory variables	*P*-value	OR	95% C.I. for OR
Restricted mobility	0.009	3.649	1.390–9.574
Experience depression	< 0.001	7.953	3.593–17.602

Area under the ROC-curve 0.708. The Hosmer and Lemeshow test: *p*-value 0.613. Nagelkerke R Square 0,211. Abbreviations: OR Odds ratio, *C.I* Confidence interval

## Discussion

The majority of participants (85%) in the present study did not experience frequent pain five years after stroke and most of the participants (64%) did not feel that they needed treatment for pain. The participants who were functionally dependent at discharge, who reported experience of depression or were restricted in their mobility had higher odds of experiencing pain frequently. Additionally, participants with more frequent pain scored their health status and recovery post-stroke significantly lower and had a poorer health-related quality of life, compared to participants who experienced less frequent pain.

Approximately 40% of the participants in the current study experienced pain to some degree (including more frequent pain as well as sometimes having pain). This is in line with findings in a previous study from Denmark [13]. A previous study in a general Swedish population reported a higher prevalence of pain, 51% [30]. The prevalence of pain both in stroke survivors and the general population has varied greatly in previous research [9, 31]. Pain is a subjective feeling and the definition of pain may vary between different individuals. Furthermore, assessing pain is difficult. For instance the wording of the questions asked may considerably influence the outcome. Several studies specifically asked about pain that started after stroke and/or pain that the participants themselves relate to their stroke [10, 13, 15, 32]. Such a distinction between pain related to stroke and pain not related to stroke has been described with a prevalence of stroke-related pain of 11% [32]. This distinction of pain was not possible in the present study, due to the design of the questionnaire, which could explain the higher prevalence of pain found in the present study.

In the explanatory regression model, restricted mobility and experienced depression were found to be significant contributors to the experience of pain. Participants who experienced depression had almost 8-fold higher odds of experiencing more frequent pain. This is in line with previous research where depression has been noted to be a significant explanatory variable for pain both in individuals with stroke [13, 33] and individuals with no prior history of stroke [15, 34]. However, the causal relationship between depression and pain remains unclear [35]. According to one review article, approximately one in three persons suffer from depression at some point post-stroke with the highest prevalence during the first year [36]. Five years post-stroke, one in five individuals fulfil the diagnostic criteria for depression [37]. In the present study, the results are based on the subjective experience of feeling depressed, which is not the same as an objective assessment or a diagnosis of clinical depression. This could explain the overall high prevalence (52%) of depression found in the current study. Participants with restricted mobility had almost 4-fold higher odds of experiencing more frequent pain (as seen in the explanatory model). Mobility restriction can be a direct consequence of stroke. Motor impairments affect approximately 80% of all stroke patients [38], and have been significantly associated with PSP [15]. Furthermore, a consequence of pain could be physical inactivity, and it could be speculated that immobility itself can cause pain, also seen in the present study. In addition to adequate pharmacological treatment, an individualized physical exercise program may therefore be an important part in pain management. Regular physical activity has been shown to decrease the intensity of pain, improve independence in activities of daily living, decrease depressive symptoms and improve range of joint movement [39, 40]. The final explanatory regression model explained 21% of the variance in the outcome which implies there are other variables not used in the present study that contribute to the experience of pain. Lower socioeconomic status, habits such as smoking and absence of personal support are all variables that have been identified as risk factors for pain in previous research and were not considered in the present study [41].

In the predictive regression model, participants that were functionally dependent at discharge had higher odds of experiencing more frequent pain 5 years post-stroke. This is in line with the results of mobility being an explanatory factor for pain, since the functional dependency scale has a substantial mobility component. However, the final model had a low rate of variance explained in the outcome and a low accuracy. Consequently, functional dependency at discharge cannot solely be used to predict who may experience more frequent pain 5 years post-stroke. These results highlight the complex nature of the pain experience and support there being multiple contributing factors in PSP. As shown previously, both demographical, premorbid and stroke-related factors have been associated with PSP [9].

In the present study, one in four participants reported unmet needs for pain treatment and 6% reported that their needs were not addressed at all. The lack of pain treatment is not only seen in the stroke population but in the general population as well. In one large-scale European study, approximately one third of the participants with chronic pain were not receiving any treatment for their chronic pain [12]. In the same study, one in five Swedish respondents reported that their current medication was inadequate in pain relief [12]. These findings highlight the need for standardized follow-up, for instance the post-stroke checklist [42], after stroke where pain is considered.

Participants who experienced more frequent pain scored their self-perceived health status and recovery post stroke significantly lower compared to the participants who experienced less frequent pain. Furthermore, more frequent pain was associated with poorer health-related quality of life. These findings further emphasise the importance of considering pain in follow-up care of all persons with stroke.

### Limitations

The generalisability of the results in the present study has some limitations. The study setting is in persons with stroke in Sweden and the social situation may not reflect the rest of the world. The study design was study based on mail-survey from a single centre cohort, lacking a control group. An age matched control group would have added information and improved the implications of the results. The majority of the participants suffered a milder stroke, which needs to be taken into account when generalizing the findings. Higher stroke severity has been associated with pain in previous research [32]. The mean age at stroke onset was 65 years which is lower than in the general Swedish stroke population. Older age has been found to be a significant explanatory variable for pain in previous research [43], which therefore could affect the generalisability of the present results.

Another limitation of the present study is that a distinction between stroke-related pain and pain not related to stroke was not possible. A questionnaire specific dedicated to distinguish different types of pain would have been beneficial for the study. However, the aim of the study was to explore experienced pain 5 years post-stroke in general, not only stroke-related pain.

The response rate was 61% in the present study. This means that 39% did not respond to the postal survey, which needs to be considered. Drop out-analysis was carried out and significantly more women were found in the non-responding group, thus yielding a study population with more men. Women overall tend to report more pain, potentially leading to a lower frequency of pain in the present study.

## Conclusion

Pain is common five years post-stroke, almost 40% of the participants reported pain of varying frequency. More frequent pain was associated with poorer quality of life and lower self-perceived health status and recovery post-stroke. Furthermore, participants who were functionally dependent at discharge, who experienced depression or were restricted in their mobility had higher odds of experiencing more frequent pain. The present study also found that one in four participants had unmet needs for pain treatment. These results emphasize the need of standardized follow-up care after stroke where persons with stroke are actively asked about pain and given appropriate treatment.

## Data Availability

The datasets analysed during the current study are not publicly available due to ethical restrictions. According to the Swedish regulation http://www.epn.se/en/start/regulations/ the permission to use data is only for what has been applied for and then approved by the Ethical board. Data are available from the authors (contact Professor Katharina S. Sunnerhagen, email: ks.sunnerhagen@neuro.gu.se) upon reasonable request.

## Abbreviations

EQ5D: EuroQol quality of life scale
H&H: Hunt & hess
ICH: Intracerebral haemorrhage
IS: Ischemic stroke
mRS: Modified rankin scale
NIHSS: National institutes of health stroke scale
PSP: Post-stroke pain
SAH: Subarachnoid haemorrhage
SALGOT: Stroke arm longitudinal study at the university of Gothenburg
SIS: Stroke impact scale
VAS: Visual analogue scale
